# Neuroimaging to monitor worsening of multiple sclerosis: advances supported by the grant for multiple sclerosis innovation

**DOI:** 10.3389/fneur.2023.1319869

**Published:** 2023-12-01

**Authors:** Jiwon Oh, Laura Airas, Daniel Harrison, Elina Järvinen, Terrie Livingston, Stefan Lanker, Rayaz A. Malik, Darin T. Okuda, Pablo Villoslada, Helga E. de Vries

**Affiliations:** ^1^Division of Neurology, St. Michael’s Hospital, Department of Medicine, University of Toronto, Toronto, ON, Canada; ^2^Department of Neurology, Johns Hopkins University, Baltimore, MD, United States; ^3^Turku PET Centre, Turku University Hospital and University of Turku, Turku, Finland; ^4^Division of Clinical Neurosciences, Turku University Hospital and University of Turku, Turku, Finland; ^5^Department of Neurology, University of Maryland School of Medicine, Baltimore, MD, United States; ^6^Baltimore VA Medical Center, VA Maryland Healthcare System, Baltimore, MD, United States; ^7^Neurology and Immunology, Medical Unit N&I, Merck OY (an affiliate of Merck KGaA), Espoo, Finland; ^8^Patient Solutions and Center of Excellence Strategic Engagement, EMD Serono, Inc., Rockland, MA, United States; ^9^Neurology & Immunology, US Medical Affairs, EMD Serono Research & Development Institute, Inc., (an affiliate of Merck KGaA), Billerica, MA, United States; ^10^Weill Cornell Medicine-Qatar, Research Division, Doha, Qatar; ^11^Institute of Cardiovascular Sciences, University of Manchester, Manchester, United Kingdom; ^12^Department of Neurology, Neuroinnovation Program, Multiple Sclerosis and Neuroimmunology Imaging Program, Clinical Center for Multiple Sclerosis, UT Southwestern Medical Center, Dallas, TX, United States; ^13^August Pi i Sunyer Biomedical Research Institute (IDIBAPS), Barcelona, Spain; ^14^MS Center Amsterdam, Department of Molecular Cell Biology and Immunology, Vrije Universiteit Amsterdam, Amsterdam Neuroscience, Amsterdam University Medical Centers (Amsterdam UMC), Location VUmc, Amsterdam, Netherlands

**Keywords:** multiple sclerosis diagnostic imaging, multiple sclerosis pathology, prognostic factors, biomarkers, machine learning

## Abstract

Key unmet needs in multiple sclerosis (MS) include detection of early pathology, disability worsening independent of relapses, and accurate monitoring of treatment response. Collaborative approaches to address these unmet needs have been driven in part by industry–academic networks and initiatives such as the Grant for Multiple Sclerosis Innovation (GMSI) and Multiple Sclerosis Leadership and Innovation Network (MS-LINK^™^) programs. We review the application of recent advances, supported by the GMSI and MS-LINK^™^ programs, in neuroimaging technology to quantify pathology related to central pathology and disease worsening, and potential for their translation into clinical practice/trials. GMSI-supported advances in neuroimaging methods and biomarkers include developments in magnetic resonance imaging, positron emission tomography, ocular imaging, and machine learning. However, longitudinal studies are required to facilitate translation of these measures to the clinic and to justify their inclusion as endpoints in clinical trials of new therapeutics for MS. Novel neuroimaging measures and other biomarkers, combined with artificial intelligence, may enable accurate prediction and monitoring of MS worsening in the clinic, and may also be used as endpoints in clinical trials of new therapies for MS targeting relapse-independent disease pathology.

## 1 Introduction

Many people with multiple sclerosis (pwMS) experience a gradual accumulation of disability in the absence of acute relapses (1). This phenomenon, known as progression independent of relapse activity or ‘silent’ progression, is evident from the earliest stages of MS (2). The underlying pathology of disease worsening independent of relapses is widespread central nervous system (CNS) damage, which is likely driven by centrally compartmentalized inflammation partially independent of peripherally mediated inflammation (3). This centrally compartmentalized inflammation is likely driven by activated microglia and astrocytes, as well as CD8+ T cells, B cells, and macrophages, whose pro-inflammatory functions are detrimental for neurons and oligodendrocytes (4). Components of this central pathology, sometimes known as smoldering MS, have been visualized using various neuroimaging techniques that measure chronic active lesions [including slowly expanding lesions and paramagnetic rim lesions (PRLs)], leptomeningeal inflammation, widespread microglial activation in normal-appearing white matter (NAWM), regional brain atrophy, and axonal degeneration. These aspects of smoldering MS are sometimes already observed early in the course of MS (5), including in radiologically isolated syndrome.

In this review, we consider recent advances in neuroimaging supported by two industry–academia collaboration programs: the Grant for Multiple Sclerosis Innovation (GMSI) and the Multiple Sclerosis Leadership and Innovation Network (MS-LINK^™^). We focus on techniques for monitoring MS pathology related to worsening and examine their potential for translation into patient care.

## 2 Unmet needs in MS

A critical unmet need in both relapsing MS and progressive MS (PMS) is the prevention of worsening, despite treatment with modern high-efficacy disease-modifying therapies (DMTs) (1). Currently approved high-efficacy DMTs target peripheral inflammation and substantially reduce the number of relapses, but fail to adequately address CNS-compartmentalized inflammation and neurodegeneration (6). MS worsening can have substantial personal and professional repercussions such as absenteeism, reduced earnings, and unemployment (7), while early intervention to reduce or delay worsening could beneficially impact the physical, mental, and cognitive well-being of pwMS. There is a limited window of opportunity when interventions may effectively stop or slow MS worsening (8), especially as most of the brain and retinal atrophy occurs in the first 5 years after disease onset (9). However, the lack of validated biomarkers for early worsening and worsening independent of relapses, together with a lack of adequately sensitive measures of remyelination and axonal regeneration, have hindered efforts to identify DMTs that could impact early MS pathology, prevent worsening, or promote recovery.

An array of novel measures of MS disease activity and worsening are being developed to more accurately measure disease worsening (10), which smoldering MS pathology likely contributes to substantially. These assessments include patient-reported outcome measures, including measures of fatigue, physical function, and quality of life; wearables and digital apps to measure cognitive domains, physical activity, and fatigue; neuropsychological assessments of cognitive, emotional, and behavioral impairment; serum and cerebrospinal fluid biomarkers of neuronal injury and reactive astrocytes; and imaging biomarkers of acute and chronic inflammation, gliosis, neurodegeneration/brain atrophy, and demyelination/remyelination (3, 10). It is not yet clear which of these alternative measures have the best prognostic value for disease worsening.

It is evident there is an ongoing need for the development of biomarkers that can detect MS worsening independent of relapses and demonstrate the impact of DMTs on disease worsening.

### 2.1 Collaborative approaches to address unmet needs in MS

#### 2.1.1 Multicenter and multinational studies

A number of research groups are currently collaborating to address these unmet needs by developing international registries and data-sharing studies. A selection of key examples is given below.

MSBase^1^ is an international online registry collecting real-world data on MS and other neuroimmunological diseases to answer epidemiological questions using demographic and clinical data from pwMS (11).

The TRaditional versus Early Aggressive Therapy for MS (TREAT-MS; ClinicalTrials.gov identifier: NCT03500328^2^) study (12) and the Determining the Effectiveness of earLy Intensive Versus Escalation Approaches for relapsing–remitting MS (DELIVER-MS; ClinicalTrials.gov identifier: NCT03535298^3^) study (12, 13) aim to guide treatment strategies and compare the impact of the traditional treatment “escalation” approach with early initiation of high-efficacy DMTs on clinical outcomes (12).

The Canadian Prospective Cohort Study to Understand Progression in MS (CanProCo) is a national cohort study that plans to follow pwMS over a 5-year period to identify biological, imaging, demographic, and clinical factors associated with disease worsening across the spectrum of MS (14).

MultipleMS and Sys4MS are European Commission–funded projects to assess the role of omics, with a special focus on genetics, in defining phenotypes and prognoses in pwMS. These projects have included studies to identify prognostic biomarkers in MS (15, 16).

#### 2.1.2 Industry–academia networks and initiatives

Collaborations between industry and academia have also made valuable contributions to address unmet needs in MS. A selection of key examples is given below.

The Progressive MS Alliance^4^ is a global collaboration involving researchers, pharmaceutical companies, non-profit organizations, healthcare professionals (HCPs), and pwMS. The alliance aims to accelerate the development of treatments for PMS by creating networks of researchers, awarding grants for research into the mechanisms of worsening, and supporting improvements in clinical trial design and implementation of trials in MS (17).

The Big Data Institute and Novartis are collaborating to use artificial intelligence (AI) to analyze large datasets from pwMS and other inflammatory diseases, with the aim of identifying early predictors of response to DMTs and have a better understanding of MS phenotypes and disease course (18).

MS Partners Advancing Technology and Health Solutions (MS PATHS^5^) is a data-sharing study sponsored by Biogen that is collecting data from pwMS to better understand MS phenotypes, support clinical decision-making, define patient outcomes, and understand the impact of demographic and clinical factors on MS prognosis (19).

The GMSI is a global initiative funded by Merck KGaA (CrossRef Funder ID: 10.13039/100009945), launched in 2013, to support academic clinical and scientific research in MS (20). Key themes of research grants awarded include non-invasive biomarkers and novel imaging techniques to monitor MS worsening, novel targets and treatments to halt disability accumulation, and improved understanding of MS pathology. The program is ongoing, but new grants are no longer awarded.

MS-LINK^™^ is a North American research network launched in 2019 and supported by EMD Serono, Inc., Rockland, MA, USA, an affiliate of Merck KGaA (CrossRef Funder ID: 10.13039/100004755) (21). It aims to deliver innovative and patient-centric research to improve outcomes in pwMS by supporting preclinical, clinical, and real-world projects addressing research priorities in the MS community.

## 3 GMSI-supported advances in neuroimaging methods and biomarkers to monitor MS worsening-related pathology

### 3.1 GMSI-supported advances in magnetic resonance imaging (MRI) methods and biomarkers to detect early MS pathology and MS worsening-related pathology

#### 3.1.1 Current use of MRI in clinical practice

**Figure 1:**
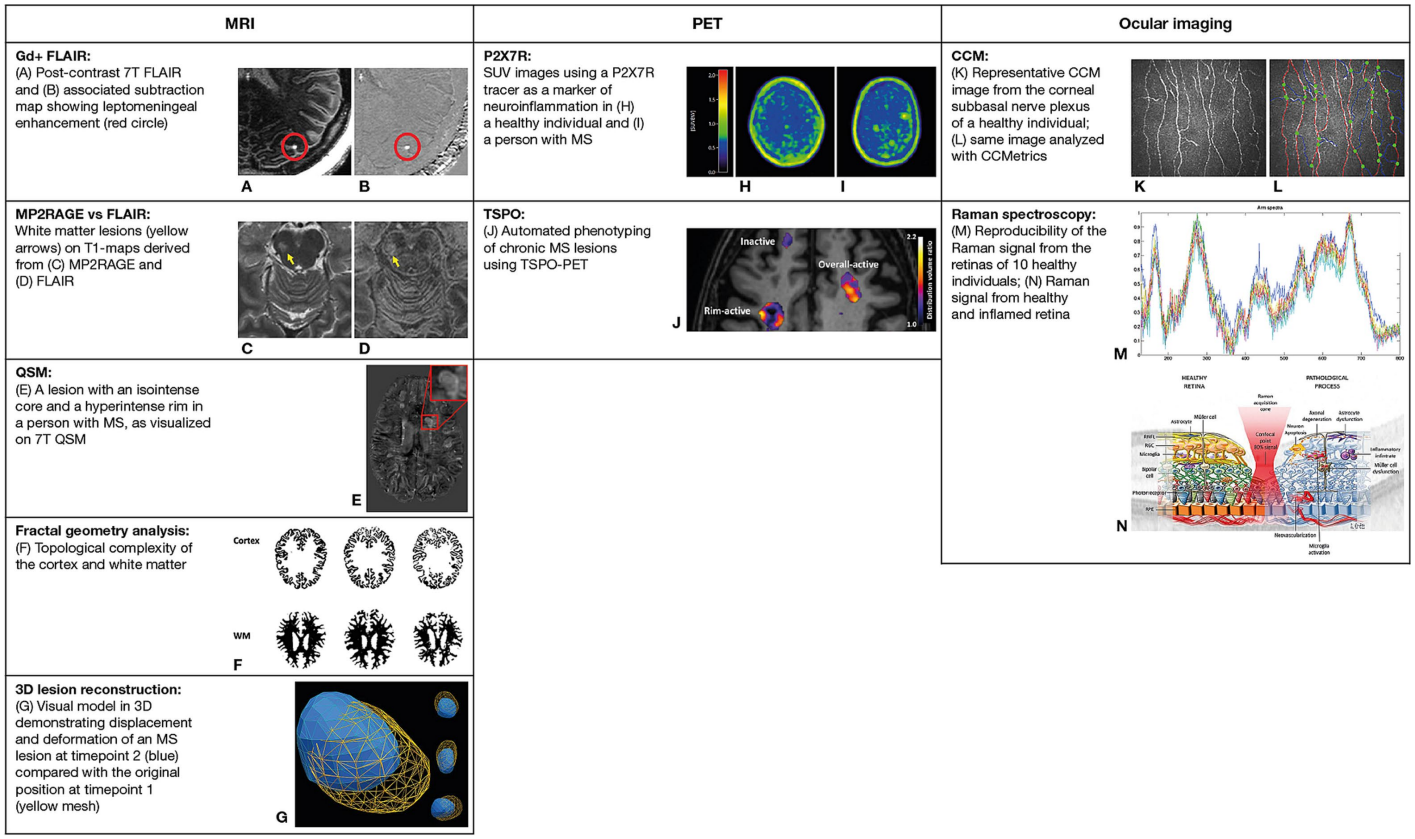
Selected neuroimaging techniques evaluated in the GMSI program. **(A,B)** Reproduced from Harrison et al., with permission of John Wiley & Sons Inc (26). **(C,D)** Reproduced from Spini et al., with permission of John Wiley & Sons Inc (27). **(E)** Reproduced from Tolaymat et al., with permission from Elsevier (28). **(F)** Adapted from Roura et al., licensed under CC BY 4.0 (34). **(G)** Reproduced from Okuda et al., licensed under CC BY 4.0 (22). **(H,I)** Adapted from Hagens et al., licensed under CC BY 4.0 (35). **(J)** Adapted from Nylund et al., licensed under CC BY 4.0 (36). **(K,L)** Reproduced from Petropoulos et al., licensed under CC BY 4.0 (37). **(M,N)** Reproduced from Alba-Arbalat et al., licensed under CC BY 4.0 (38). 7T, 7-Tesla; CCM, corneal confocal microscopy; FLAIR, fluid-attenuated inversion recovery; Gd+, gadolinium-enhanced; GMSI, Grant for Multiple Sclerosis Innovation; MP2RAGE, magnetization-prepared 2 rapid acquisition gradient echo; MRI, magnetic resonance imaging; MS, multiple sclerosis; PET, positron emission tomography; QSM, quantitative susceptibility mapping; R, receptor; SUV, standardized uptake value; TSPO, translocator protein; WM, white matter.

**Figure 2:**
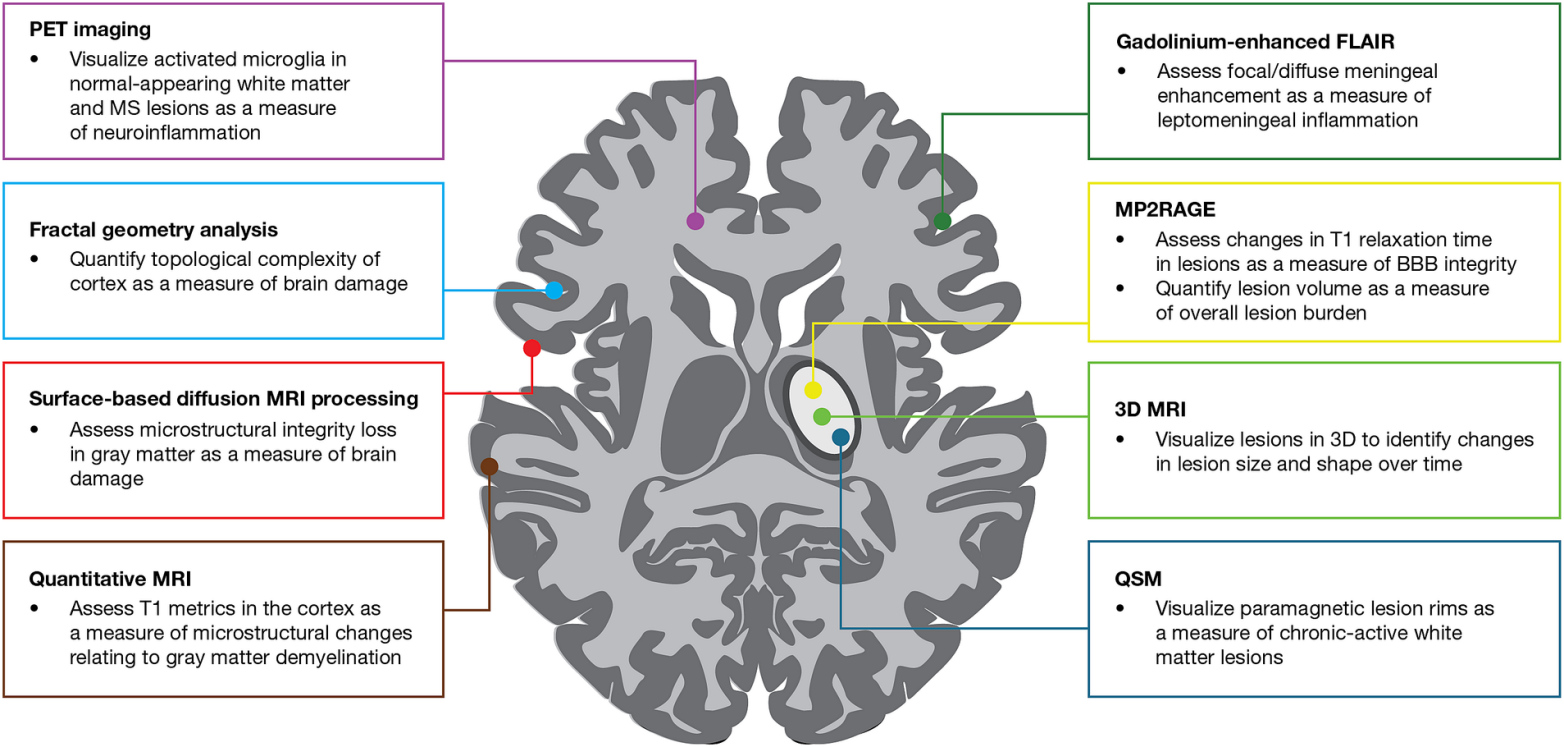
Use of MRI and PET imaging techniques evaluated in the GMSI program for detecting MS worsening-related pathology. BBB, blood–brain barrier; FLAIR, fluid-attenuated inversion recovery; GMSI, Grant for Multiple Sclerosis Innovation; MP2RAGE, magnetization-prepared 2 rapid acquisition gradient echo; MRI, magnetic resonance imaging; MS, multiple sclerosis; PET, positron emission tomography; QSM, quantitative susceptibility mapping.

Conventional, clinical-grade MRI to identify new/enlarging T2-hyperintense or gadolinium-enhancing lesions is currently the standard of care for monitoring MS disease activity in clinical practice. In some centers, where relevant technology is available, clinical-grade MRI can also be used to detect chronic lesions, and changes in regional and whole brain volume, but these measures are not widely applied in clinical decision-making. Conventional MRI is limited by a lack of sensitivity and specificity to tissue microstructural changes, including those associated with disease worsening (3). Here, we describe recent advances in the application of MRI to address unmet needs in the detection of early MS pathology and worsening, supported by the GMSI program [Table 1, Figure 1 (22, 26–28, 34–38), and Figure 2].

**Table 1:** Key features of selected MRI techniques evaluated in the GMSI program for detecting MS worsening-related pathology.

Technique	Key capabilities	Limitations	Clinical applicability
3D lesion reconstruction (22–25)	View lesions in 3D Assess dynamic changes in lesion size and shape	Limited to focal lesions, rather than T2-hyperintensities that coalesce over time	May enable identification of lesion deformation and displacement not visible on 2D images which may be relevant to diagnosis, prognosis, and treatment response
7T MRI (26–32)	Increases in resolution and signal-to-noise ratio Wider metabolic spectra and greater susceptibility effects Ability to resolve microstructural changes Assess T1 metrics	Suboptimal signal-to-noise ratio and signal dropout in lower parts of brain and brain-skull interfaces Field inhomogeneity Expense of purchasing and installing additional scanners Application to the cortex at 3T is limited by partial volume averaging of the thin cortex with adjacent tissues	Increased sensitivity for visualization of lesions, meningeal enhancement, etc. Measurement of T1-relaxation-time changes in lesions, including changes induced by gadolinium Increased resolution enables identification of changes in individual cortical layers that are not apparent across the full cortical thickness qMRI images may be more reproducible than weighted images
Gadolinium-enhanced FLAIR (27, 30)	View lesions and focal/diffuse leptomeningeal enhancement	Meningeal enhancement is not specific to MS – also seen in meningeal infection and carcinomatosis Use at 7T results in suboptimal signal-to-noise ratio and heterogenous T1 weighting	Non-invasive method to quantify persistence of meningeal inflammation
MP2RAGE (27, 31)	Assess BBB integrity through changes in T1 relaxation time Quantify lesion volume	Not yet known whether BBB changes reflect lesion formation or chronic inflammation	Simultaneous acquisition of all necessary images reduces scan times compared with spoiled gradient-echo or other T1-relaxation-time measurement methods Simple analysis, unlike dynamic contrast-enhanced MRI permeability measurements
QSM (28)	View paramagnetic rim lesions	Complex post-acquisition processing Paramagnetic rim lesions are not present in all pwMS	Can be performed at 3T
Surface-based diffusion MRI processing (33)	Assess microstructural integrity loss in gray matter	Not specific to MS – similar changes seen in people with Alzheimer’s disease and frontotemporal dementia	May be more sensitive than volume loss as a measure of cortical damage
Fractal geometry analysis (34)	Monitor topological complexity of the brain	Not specific to MS – similar changes seen in people with dementia and those who have had a stroke	Can be calculated from standard 3TT1 images with no need for additional scanners or sequences

For many of the techniques detailed above, the technology required to process images is complex and not readily available.

3T, 3-Tesla; 7T, 7-Tesla; BBB, blood–brain barrier; FLAIR, fluid-attenuated inversion recovery; GMSI, Grant for Multiple Sclerosis Innovation; MP2RAGE, magnetization-prepared 2 rapid acquisition gradient echo; MRI, magnetic resonance imaging; MS, multiple sclerosis; pwMS, people with MS; q, quantitative; QSM, quantitative susceptibility mapping.

#### 3.1.2 Measurement of 3D conformational characteristics to accurately identify MS lesions and effectively monitor disease activity

3D lesion conformational characteristics include shape, texture, structure, and surface patterns, as well as the evolution of these characteristics over time. These characteristics were found to differ between MS lesions and lesions originating from other conditions such as non-specific white matter (WM) disease, small vessel disease, or neuromyelitis optica spectrum disorder (22, 23, 39, 40). MS lesions were more likely to be asymmetrical than non-MS lesions, with a complex surface morphology and a multi-lobular elongated shape (39). Over time, MS lesions had greater shifts in 3D displacement and became less spherical than non-MS lesions (22). Differences in 3D displacement between types of MS lesions were also identified: slowly expanding lesions moved toward the cortex, while lesions that decreased in size moved toward the center of the brain (23). Changes in 3D conformational characteristics of T2 lesions that evolve to T1 hypointensities may offer insights into the pathobiology of injury, treatment effects, and measures that better predict future risk of disability worsening. Additionally, 3D conformational measures may enable the identification of regions within the CNS that are selectively vulnerable to injury (40).

Conventional, 2D, clinical-grade MRI limits the amount of information accessible to HCPs, restricting their ability to diagnose and monitor MS (24). The study of 3D conformational characteristics and dynamic changes over time may provide additional information on MS worsening, stability, or improvement, and enable the assessment of treatment impact (24) to support HCP decision-making regarding treatment optimization. To effectively translate 3D lesion reconstruction into the clinic, further studies are required to define the relationships between changes in conformational characteristics and long-term clinical outcomes (22).

#### 3.1.3 Multimodal MRI at 7-Tesla (7T) to interrogate chronic inflammation and MS pathology

Ultra-high field, 7T MRI can provide higher-resolution images than clinical-grade MRI, allowing for greater sensitivity and precision when investigating MS pathology. Here, we describe the application of this technique to the investigation of meningeal inflammation, blood–brain barrier (BBB) disruption, PRLs, and cortical tissue.

Meningeal inflammation in MS is thought to induce changes in cortical microglia that are associated with neurodegeneration (41). Widespread meningeal inflammation has been reported in histopathological studies of MS, but imaging of this inflammation has proved challenging due to insufficient resolution and sensitivity of conventional MRI enhancement (26). Using 7T MRI, researchers identified diffuse enhancement in the subarachnoid space on gadolinium-enhanced, 3D-fluid-attenuated inversion recovery (3D-FLAIR) in the majority of participants with MSwhich was associated with reduced cortical gray matter volume (26). The study population was relatively stable and only moderately disabled (median EDSS was 3.0), consisting of mainly RRMS (72%), but also SPMS (14%) and PPMS (14%) subjects. In contrast, nodular foci were present both in healthy volunteers and pwMS, suggesting that nodular foci may be a normal variant, while diffuse enhancement may represent breakdown of the blood–meningeal barrier at sites of meningeal inflammation (26). Further, leptomeningeal enhancement was associated with cortical thickness but not with focal cortical lesions, suggesting that meningeal inflammation may trigger more widespread neurodegeneration rather than focal demyelination (29). Persistence of enhancement in the leptomeningeal space was observed at 1 and 2 years of follow-up (30); persistence, particularly of diffuse enhancement through the subarachnoid space at 1 year, was associated with disability worsening as assessed by Expanded Disability Status Scale (EDSS) scores (30). There were no significant differences in persistence between pwMS receiving DMTs and untreated pwMS, suggesting that currently approved DMTs may not adequately control meningeal inflammation (30).

Ultra-high field MRI techniques may also allow for more precise measurements of persistent BBB disruption in MS lesions beyond the clearly evident gadolinium enhancement that occurs in acute lesions (31). A magnetization-prepared 2 rapid acquisition gradient-echo (MP2RAGE) sequence was used to assess gadolinium-induced T1 signal change (measured by change in T1 relaxation time, termed ΔT1 in the study) in non-enhancing lesions and NAWM after contrast administration (31). While visible contrast enhancement could not be observed on T1-weighted images for the non-enhancing lesions, a gadolinium-induced ΔT1 was measured and was greater in non-enhancing lesions than in NAWM, and correlated with increased disability (31). This suggests that gadolinium-induced ΔT1 may be a biomarker for persistent BBB disruption and possibly chronic WM lesion inflammation (31). MP2RAGE enables simultaneous acquisition of all necessary images, resulting in reduced scan times, and analysis is simpler than complex modeling of dynamic contrast-enhanced MRI permeability measurements; therefore, MP2RAGE may be more clinically feasible compared with spoiled gradient-echo or other T1-relaxation-time measurement methods (31).

Susceptibility-weighted phase imaging and quantitative susceptibility mapping (QSM) can be used to identify chronic active lesions by the presence of paramagnetic rims (28). The clinical relevance of this finding was evaluated through the GMSI program in a study where QSM images from 7T multi-echo gradient echo revealed that male pwMS had a greater burden of PRLs (28). This suggests that the interplay between the innate immune system and MS pathology may differ based on biologic sex (28).

The high resolution of 7T MRI has also been employed to investigate the thin and highly folded tissue of the cortex in detail. Quantitative MRI (qMRI) derived from 7T images was used to investigate relationships between T1 metrics in individual cortical layers and Symbol Digit Modalities Test (SDMT) scores (32). This revealed layer-specific relationships between T1 metrics and disability that were not apparent when qMRI values were summarized across the full thickness of the cortex (32).

Although 7T provides greater resolution than 3T, use of FLAIR at 7T has limitations, including suboptimal signal-to-noise ratio and heterogenous T1 weighting, resulting in indistinct lesion borders and difficulty distinguishing lesions from noise (27). MP2RAGE and FLAIR at 7T were compared for their ability to quantify WM lesion volume; while both showed similar relationships with disability scores, MP2RAGE demonstrated superior image noise and resolution of lesion borders than FLAIR (27).

The clinical applicability of these advanced techniques depends greatly on their accessibility. Technologies such as 7T MRI are not widely available at the current time due to equipment cost, and they may therefore be of more use in research settings than in clinical practice. Insights provided by the application of 7T MRI to investigate MS pathology may guide further research efforts in this field, such as considering meningeal inflammation as an endpoint in clinical trials of new therapies for MS, since this does not appear to be adequately controlled by current DMTs (30). Clinical trial development and analysis may also be informed by knowledge of the male predominance of PRLs (28).

#### 3.1.4 Surface-based diffusion MRI processing to assess microstructural changes in MS

Gray matter damage is thought to play an important role in MS worsening, but imaging of microstructural gray matter damage across the different stages of MS has proved challenging (33). Using a surface-based diffusion MRI processing tool, patterns of microstructural integrity loss were found to differ between early and late MS (33). Changes in diffusivity were apparent within the first 5 years of disease onset, and increased diffusivity was associated with increased disability (33). However, areas of diffusivity change did not overlap with areas of volume loss during early MS; therefore, diffusivity was identified as a potentially more sensitive measure of gray matter damage than volume loss in early MS (33).

#### 3.1.5 Fractal geometry analysis of brain MRI to predict disability worsening

A clinically accessible method to monitor tissue damage in the brain may lead to improvements in treatment decision-making in MS. Fractal dimension assesses the topological complexity of the brain and captures the effect of CNS damage (34). Using 3D T1-weighted magnetization-prepared rapid acquisition gradient echo at 3T, reduced fractal dimension of the cortex was found to predict disability worsening over a 5-year period, as assessed by EDSS, 9-Hole Peg Test, SDMT, Sloan 2.5% contrast cards, and MS Functional Composite-4 (34). Additionally, thresholds of fractal dimension were identified as potential predictors for the risk of disability worsening in the short-to-medium term (up to 5 years). If validated in larger groups of patients, it is possible that reduced fractal dimension could act as a prognostic marker for MS worsening and help to identify pwMS who may benefit from switching to a higher-efficacy DMT (34).

As fractal dimension of the brain can be calculated from standard T1 sequences at 3T, it has greater potential than more complex techniques to be readily applied in clinical settings without the need for additional scanners or specialized sequences. However, a limiting factor of more readily accessible techniques is the need for technical expertise with post-acquisition processing to yield quantitative measures, and a lack of guidance for integrating these measures into clinical decision-making.

### 3.2 GMSI-supported advances in application of positron emission tomography (PET) to characterize central inflammation and detect worsening-related pathology

#### 3.2.1 Current use of PET

Current PET tracers target the translocator protein (TSPO) to characterize neuroinflammation, as TSPO is overexpressed in activated microglia (42). However, TSPO cannot discriminate between pro- and anti-inflammatory activated microglial states (43), both of which might play a role in MS pathogenesis; hence, it is most informative to interpret TSPO-PET imaging findings from brains of pwMS in the context of clinical data, and in comparison to results from age- and sex-controlled healthy individuals. Here, we describe recent advances supported by the GMSI program in the application of TSPO-PET to investigate CNS pathology in MS *in vivo*, and discuss how TSPO-PET can be used to predict disease worsening in MS. We also look into the GMSI-supported identification of new PET targets and tracers to image various microglial phenotypes [Table 2, Figure 1 (22, 26–28, 34–38), and Figure 2].

**Table 2:** Key features of selected PET imaging techniques evaluated in the GMSI program for detecting MS worsening-related pathology.

Technique	Key capabilities	Limitations	Clinical applicability
TSPO-PET (44–47)	Identify activated microglia	Cannot distinguish between pro-inflammatory and anti-inflammatory microglia TSPO may also be expressed by non-microglial cells Tracer binding affinity varies between individuals due to a polymorphism on the TSPO gene	May enable clinical trial screening of pwMS at risk of worsening
P2X7R-PET (35, 47, 48)	Identify pro-inflammatory microglia	Pro-inflammatory microglia are not specific to MS – similar changes are seen in people with other neuroinflammatory diseases	Specific for pro-inflammatory microglia, unlike TSPO May enable clinical trial screening of pwMS at risk of worsening

GMSI, Grant for Multiple Sclerosis Innovation; MS, multiple sclerosis; PET, positron emission tomography; pwMS, people with MS; R, receptor; TSPO, translocator protein.

#### 3.2.2 TSPO-PET detection of microglial activation in the brain in association with disease progression

The best value and promise of TSPO-PET imaging lies in its ability to quantify smoldering inflammation in the brains of pwMS (44, 49), where certain characteristics correlate with cognitive impairment in even the earliest stages of MS (50). TSPO-PET studies have demonstrated increased widespread microglial activation in NAWM, in normal-appearing gray matter, and the thalamus in secondary progressive MS (SPMS), compared with in relapsing–remitting MS (RRMS) (36, 44, 49). Additionally, TSPO-PET can be used to phenotype chronic lesions based on innate immune cell activation at the edge of the lesion. A higher proportion of TSPO-PET–detectable chronic active lesions were observed in people with SPMS compared with RRMS (36). Importantly, TSPO binding in the thalamus, NAWM, and perilesional NAWM predicted future MS worsening independent of relapses (44, 51). TSPO-PET studies on several approved DMTs have demonstrated a reduction in microglial activation, either in focal lesions (52–54) or in the NAWM (54), and a longitudinal study in untreated SPMS showed a TSPO-PET–measurable increase in microglial activation in the perilesional NAWM (54).

TSPO-PET imaging has potential for use in clinical trials to facilitate selection of pwMS who are more likely to experience worsening, and to measure neuroinflammatory outcomes. PET imaging of microglia may also be useful in the clinic to identify pwMS who may benefit from a DMT that targets the innate immune system. However, TSPO-PET is not widely applicable in the clinic due to the highly demanding technology required, and complexities in image analysis and modeling. Increased availability and standardization of analysis pipelines for TSPO binding may mitigate this issue. More easily measurable markers are still needed to identify and differentiate microglia and macrophage activation.

#### 3.2.3 PET imaging of microglial phenotypes

TSPO-PET imaging has some limitations, such as the TSPO gene polymorphism-driven heterogeneity in the binding affinity of the second-generation TSPO ligands, and TSPO expression by cell types other than microglia, including macrophages, a subset of astrocytes, and some endothelial cells (45, 55, 56). Increased TSPO binding in MS lesions is considered to be explained by microglial activation; one manifestation of this is translocation of these cells into areas of damage or inflammation, which leads to increased microglial density (46). Due to limitations in the ability of TSPO ligands to differentiate between the various innate immune cell phenotypes, new PET imaging targets to identify differential microglial activation have been evaluated (4). Two potential new PET imaging targets were identified: P2X7 receptor (P2X7R) and P2Y12 receptor (P2Y12R) (47). P2X7R was found to be associated with pro-inflammatory microglia in MS lesions and the disease peak in experimental autoimmune encephalomyelitis (EAE), an inflammatory animal model of MS. P2Y12R was associated with more homeostatic microglia in NAWM in post-mortem brains from pwMS and remission in an EAE animal model (41, 47).

Following the identification of P2X7R as a potential target for PET imaging, it was noted that new tracers were needed to enable evaluation and validation of P2X7R in animal models, as currently available P2X7R tracers have low BBB permeability or low affinity to rodent P2X7R (48). The radioligand [11C]SMW139 was identified as having good characteristics as a tracer for P2X7R to image neuroinflammation and pro-inflammatory microglia, with acceptable binding affinity in both rats and humans (48). A first-in-human study confirmed that [11C]SMW139 could be used as a tracer to identify neuroinflammation in MS lesions and normal-appearing brain tissue in RRMS (35).

### 3.3 GMSI-supported advances in ocular imaging methods and biomarkers to detect MS worsening-related pathology

#### 3.3.1 Current use of ocular imaging

**Figure 3:**
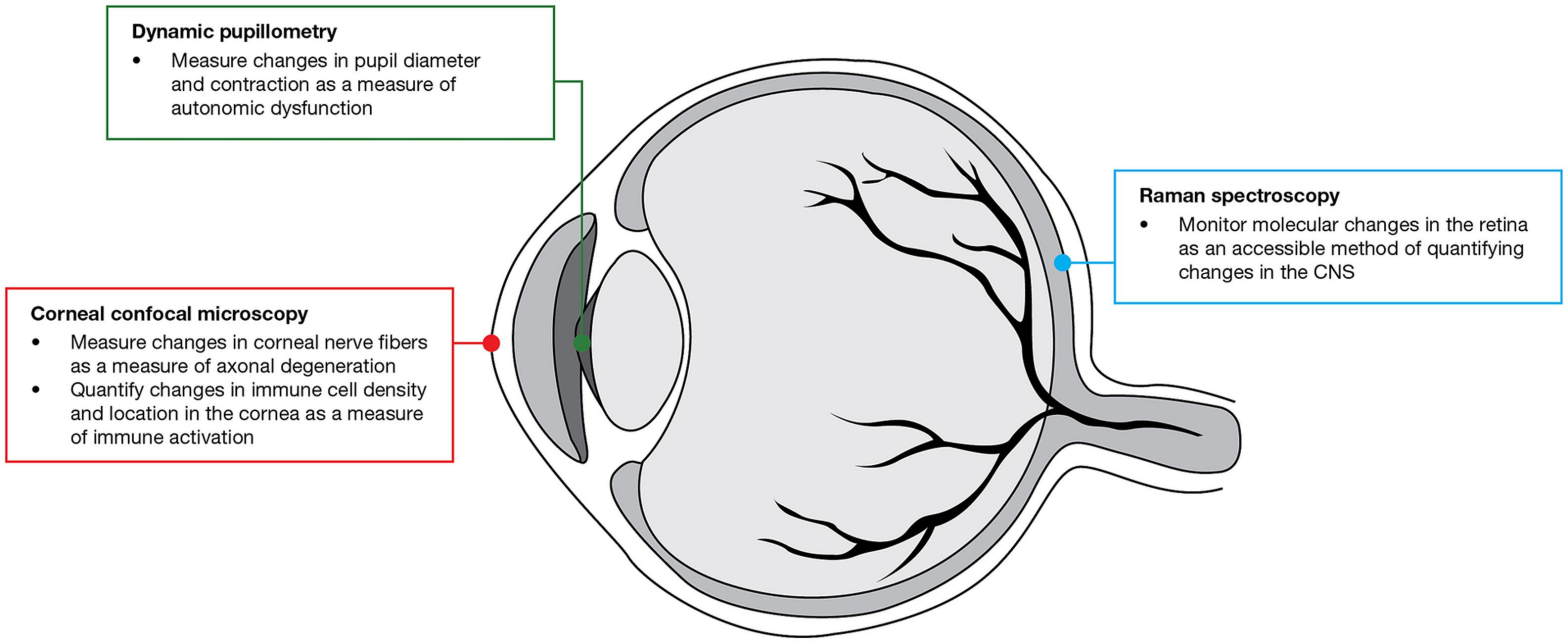
Use of ocular imaging techniques evaluated in the GMSI program for detecting MS worsening-related pathology. CNS, central nervous system; GMSI, Grant for Multiple Sclerosis Innovation; MS, multiple sclerosis.

MS commonly manifests clinically with visual symptoms, and the retina is a relatively accessible portion of the CNS, making ocular imaging an attractive option to investigate central pathology. Optical coherence tomography (OCT) imaging has been used to measure retinal nerve fiber layer thickness as a surrogate for axonal loss (57), and the thickness of the ganglion cell/inner plexiform layer of the retina has been shown to predict disability worsening (58). However, retinal nerve fiber layer thickness can be affected by acute optic neuritis, which is common in pwMS, limiting its value in identifying long-term worsening and evaluating the effect of therapeutic interventions (57, 58). Here, we describe recent advances in ocular imaging to predict MS worsening, supported by the GMSI program [Table 3, Figure 1 (22, 26–28, 34–38), and Figure 3].

**Table 3:** Key features of selected ocular imaging techniques evaluated in the GMSI program for detecting MS worsening-related pathology.

Technique	Key capabilities	Limitations	Clinical applicability
Corneal confocal microscopy (37, 57, 59, 60)	Measure changes in corneal nerve fibers Quantify changes in immune cell density and location	Manual quantification of images is time consuming and subjective	Not affected by optic neuritis Automated quantification is less time consuming and reduces bias Non-invasive objective quantification of axonal degeneration
Dynamic pupillometry (61)	Measure changes in pupil diameter and contraction	Not specific to MS – similar changes have been seen in people with diabetes, Alzheimer’s disease, and an overactive bladder	Independent of previous optic neuritis Simple and non-invasive measure of autonomic dysfunction
Raman spectroscopy of the retina (38)	Monitor molecular changes in the retina	Molecular signals averaged across retinal layers prevents identification of specific layer or cell changes	Non-invasive monitoring of the CNS, enabling early diagnosis and prognosis

CNS, central nervous system; GMSI, Grant for Multiple Sclerosis Innovation; MS, multiple sclerosis.

#### 3.3.2 Corneal imaging for prediction of MS worsening

Based on the limitations of OCT, there was a need for a new surrogate endpoint to identify early axonal degeneration and accurately predict worsening of MS. Corneal confocal microscopy (CCM) has been used to identify corneal nerve fiber loss, which is not affected by optic neuritis, age, or MS duration (Table 3) (57). A decrease in corneal nerve branch density was associated with disease severity based on EDSS score and MS Severity Score (57). A subsequent longitudinal study showed that a reduction in corneal nerve fiber density, length, width, and area correlated with disability worsening over 2 years in people with RRMS (59).

In addition to identifying loss of corneal nerve fibers as a surrogate for axonal degeneration in MS, CCM can also be used to image antigen-presenting immune cells in the cornea as a measure of immune activation (59, 60). Indeed, increased dendritic cell density was found to correlate with disability worsening over 2 years in people with RRMS (59). Furthermore, in pwMS, there was an increase in immature immune cell density and the distance between these cells and nerve fibers, compared with healthy participants (60). Quantification of changes in corneal immune cells may act as a biomarker for MS disease status and the effect of DMTs.

A perceived limitation of CCM was the need for manual quantification of corneal nerve morphology, which is time consuming and subjective (37). However, machine-learning algorithms for rapid automated quantification have recently been used to identify corneal axonal loss in people with RRMS, SPMS, and clinically isolated syndrome (37). Furthermore, automated corneal nerve quantification enables standardized and unbiased assessment in multicenter trials of new therapies for MS.

Unlike OCT, CCM measurements are not influenced by optic neuritis, making assessment of long-term worsening applicable even in pwMS who have recently experienced acute optic neuritis (57).

#### 3.3.3 Dynamic pupillometry for detection of autonomic dysfunction in MS

Dynamic pupillometry was investigated as a potential tool to assess autonomic dysfunction in MS (61). The initial pupil diameter and pupil contraction amplitude in response to light were significantly lower in pwMS compared with healthy participants (61). The initial pupil diameter correlated with EDSS score, and the pupil contraction latency correlated with retinal nerve fiber layer thickness, independent of previous optic neuritis, enabling the assessment of long-term worsening even in pwMS with acute optic neuritis (61). These findings are of interest as pupillometry is a relatively simple clinical tool with applications in a wide range of settings, but further validation in larger groups of patients is needed before clinical application.

#### 3.3.4 Raman spectroscopy of the retina and inflammation in MS

The monitoring of molecular changes in the CNS using PET, magnetic resonance spectroscopy, or near-infrared imaging is limited by the spatial and temporal resolution of these technologies and the number of molecules that they can assess. Raman spectroscopy of the retina was identified as a potential method to more comprehensively quantify molecular changes in a physically accessible part of the CNS (38). Using Raman spectroscopy, changes in the metabolic profile of the retina were identified in pwMS, compared with healthy participants. Both acute and chronic inflammation were found to be associated with changes in levels of molecules relating to metabolism, excitotoxicity, and maintenance of neurons and synapses. Alterations in amyloid-β and α-synuclein were identified as potential biomarkers of CNS degeneration (38). Although still in the early stages of investigation, with further studies needed, molecular imaging of the retina may enable identification of early biological changes before loss of structural and functional integrity occurs, as well as identification of subgroups of pwMS with closely related pathogenesis, which may help HCPs predict prognosis or response to therapy.

### 3.4 GMSI-supported advances in the application of machine learning with neuroimaging to predict MS worsening

#### 3.4.1 Current use of machine learning and novel analytic methods

The advances in neuroimaging technology described above aim to identify or apply predictive markers of MS disease severity or treatment response. Machine learning may have the potential to support these efforts by assessing correlations between markers individually or in combination, and in current or future disease states (62).

#### 3.4.2 Machine learning and neuroimaging measures to predict cognitive impairment

Machine learning was applied to combinations of imaging and non-imaging measures to predict cognitive impairment in MS (63). Several machine-learning models were created and validated using training and test datasets. The most representative model was selected; this model identified educational level, disease severity, lesion burden, and hippocampus and anterior cingulate cortex volume as the best predictors of cognitive impairment (63).

With further study and validation across different MS populations, machine-learning models may help to identify pwMS at risk of cognitive impairment, enabling targeting of support programs and activities, including cognitive rehabilitation and intellectual enrichment, that may attenuate the impact of MS on cognition (63). Machine learning may also enable the identification of combinations of biomarkers predictive of MS worsening, which may allow for personalized treatment strategies to maximize improvements in clinical outcomes in pwMS over time.

## 4 Future directions

### 4.1 Ongoing MS-LINK^™^–supported research aiming to develop neuroimaging methods and biomarkers to detect early MS worsening and related pathology

Ongoing research projects continue to apply advances in neuroimaging technology to detect MS worsening-related pathology and the assessment of treatment impact. The MS-LINK^™^ real-world data and patient-reported outcome network is collaborating with the University of Texas Southwestern Medical Center to use MRI, 3D visual modeling, and AI for the improvement of MS diagnosis and monitoring of disease activity, and to assess the impact of DMTs on lesion size, shape, and surface features.

The MS-LINK^™^ scientific and precision-medicine network is collaborating with the National MS Society of the USA and the Myelin Repair Foundation to determine how neuroimaging biomarkers of relapse-independent disease pathology correspond with innovative blood biomarkers. Blood biomarkers may be more clinically accessible than neuroimaging technologies, as blood sample collection is rapid and may be less costly than acquiring imaging studies, which require expensive equipment and additional resources. Ongoing projects include the investigation of levels of CNS-derived extracellular vesicles in blood as biomarkers of chronic active lesion activity in MS. One early output of this collaboration is a report where measurements of mitochondrial activity in neuronally enriched extracellular vesicles predicted brain and retinal atrophy in MS (64).

### 4.2 Ongoing research gaps

**Figure 4:**
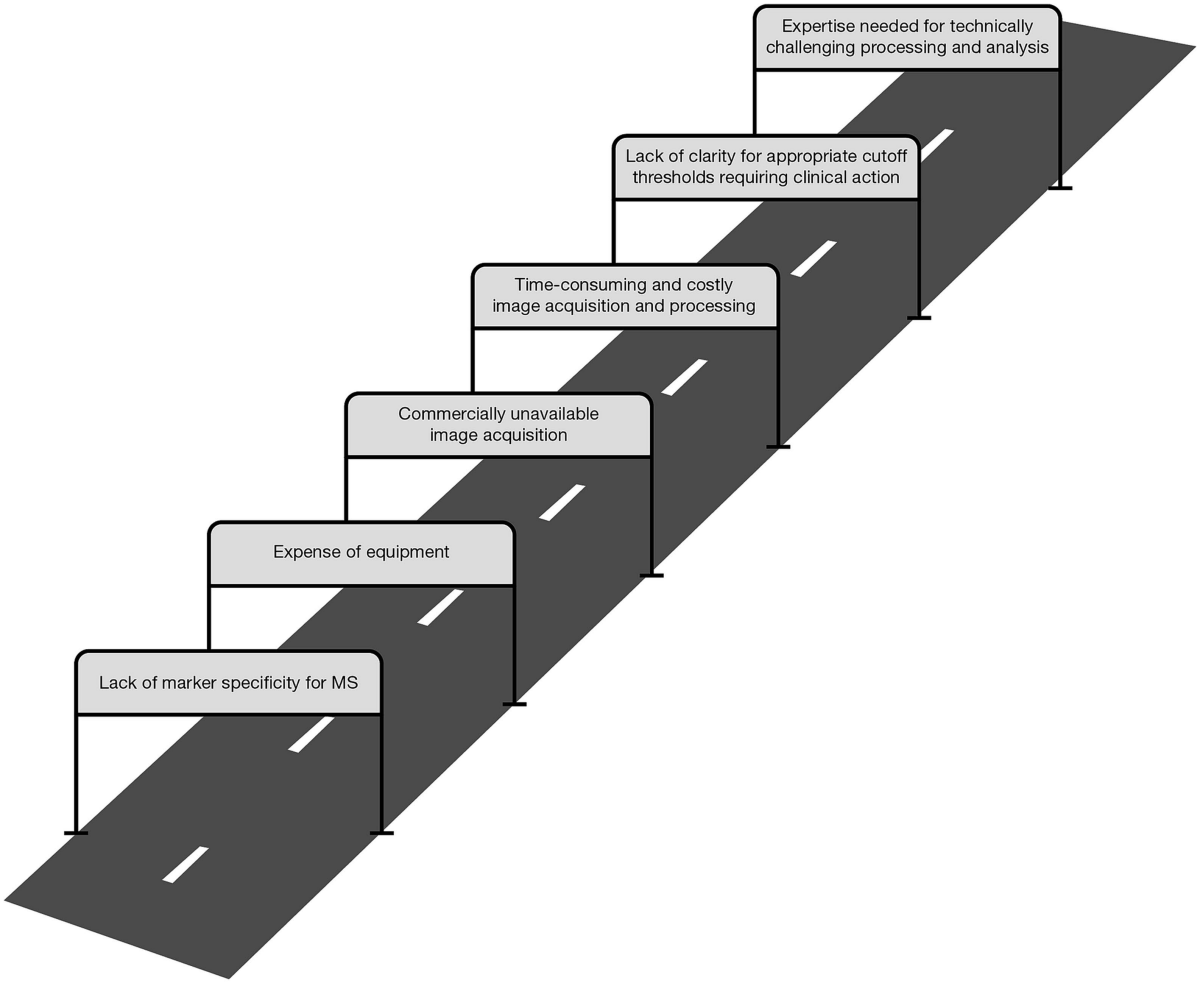
Hurdles faced by MS neuroimaging measures in translation from the laboratory to the clinic. MS, multiple sclerosis.

There remains a need to identify and develop additional neuroimaging methods and biomarkers to identify early MS pathology and worsening, for use in both clinical trials to rapidly evaluate new therapies and in the clinic to provide optimal care for pwMS. Methods and biomarkers that have already been developed require validation before they can be translated and applied to clinical use. Clinical availability of developed tools is a major gap, due to factors such as expense of equipment or complexity of techniques (Figure 4). Due to HCP time constraints, minimal acquisition and post-processing times are necessary for clinical use.

There also remains a need for DMTs capable of preventing worsening independent of relapses. Novel therapies, including Bruton’s tyrosine kinase inhibitors, several of which are currently in Phase III clinical trials for MS, aim to tackle worsening by targeting chronic neuroinflammation in the CNS (65), and the results of these clinical trials are eagerly anticipated. Beyond the prevention or delay of worsening, treatments that may promote remyelination and disability improvement are also required. Neuroprotective and regenerative therapies to prevent CNS damage and promote functional recovery may include drugs, gene therapy, and cell therapy, which could be applied in combination with currently approved DMTs that target acute inflammation and relapses (3, 66).

## 5 Conclusion

Collaborative approaches addressing unmet needs in MS, including collaborations between industry and academia such as the GMSI and MS-LINK^™^ programs, have resulted in valuable advances in our understanding of MS. GMSI-supported advances in neuroimaging methods and biomarkers to identify early pathology and worsening in MS include developments in the application of MRI, PET, ocular imaging, and machine learning, which are summarized above.

Advances in neuroimaging measures face hurdles in translation from the laboratory to the clinic. These often relate to the expense of required equipment and the time, cost, and technical expertise required to perform and process the relevant techniques and data. In addition, determining appropriate thresholds that require clinical action are often unclear with various quantitative measures. Some of these hurdles may be overcome through automation and machine-learning approaches; however, other measures may be more suitable exclusively for research use. A greater understanding of MS worsening-related pathology and the ways in which neuroimaging and non-imaging measures are related may also allow for the use of more accessible measures, such as blood biomarkers, as surrogate markers for more complex measures of worsening. In cases where measures are not sufficiently accessible for use in standard clinical practice, it may still be possible to use them as clinical trial endpoints of worsening independent of relapses, providing expedited evidence of treatment efficacy to facilitate regulatory approval of novel DMTs.

In the future, ongoing research may enable the use of combinations of neuroimaging measures, non-imaging biomarkers, and AI to predict and identify worsening in clinical practice more accurately to provide personalized treatment for pwMS. Such efforts could support individual treatment decision-making to provide pwMS at risk of disease worsening with more targeted therapies to maximize treatment outcomes in the long term.

## Scope statement

This review focuses on the application and potential for translation of recent select advances in neuroimaging to quantify disease worsening and related pathology in multiple sclerosis (MS). Most people with MS gradually experience worsening disability, despite high-efficacy therapies that reduce relapses. To identify therapies that can prevent this worsening, there is a need to develop techniques that can predict and monitor disability worsening and detect related pathology. Industry–academia collaborations such as the Grant for Multiple Sclerosis Innovation (GMSI) and Multiple Sclerosis Leadership and Innovation Network (MS-LINK™) programs are two initiatives striving to address these unmet needs. Our review highlights the importance of industry–academia collaborations and reviews developments in the application of select neuroimaging techniques for predicting and monitoring disability worsening in MS. The possibilities and challenges with translation of these techniques through to patient care, ongoing research, and future directions that may enable the provision of more optimal care for people living with MS are also discussed.

## Author contributions

JO: Conceptualization, Writing – review & editing. LA: Writing – review & editing. DH: Writing – review & editing. EJ: Conceptualization, Funding acquisition, Writing – review & editing. TL: Conceptualization, Writing – review & editing. SL: Conceptualization, Funding acquisition, Writing – review & editing. RM: Writing – review & editing. DO: Writing – review & editing. PV: Writing – review & editing. HV: Writing – review & editing.

## Footnotes

^1^ https://www.msbase.org/

^2^ https://treat-mstrial.org/

^3^ https://deliver-ms.com/

^4^ https://www.progressivemsalliance.org/

^5^ https://www.mspaths.com/
